# The Time Delay Between Drug Intake and Bronchospasm for Nonsteroidal Antiinflammatory Drugs Sensitive Patients

**DOI:** 10.1097/WOX.0b013e3181fdfc5f

**Published:** 2010-12-15

**Authors:** Giedre Grigiene, Jolita Norkūnienė, Violeta Kvedariene

**Affiliations:** 1 Vilnius University, Faculty of Medicine, Lithuania; 2 Vilnius University Emergency hospital, Lithuania; 3 Vilnius Gediminas Technical University, Faculty of Fundamental Sciences, Lithuania; 4 Vilnius University hospital Santariskiu klinikos, Lithuania

**Keywords:** hypersensitivity to nonsteroidal antiinflammatory drugs, bronchospasm, asthma, aspirin

## Abstract

A study was performed to assess the time between drug intake and drug induced hypersensitivity reaction for patients sensitive to nonsteroidal antiinflammatory drugs (NSAID) in clinical patient history and after oral provocation tests. Drug hypersensitivity ENDA questionnaires were filled for the patients with suspected sensitivity to NSAID. Oral provocation tests were performed with suspected NSAID according to the ENDA/EAACI recommendations. There were 76 patients with history of hypersensitivity reactions after use of NSAID enrolled in the study. Recorded were 154 hypersensitivity reactions to NSAID in the clinical history. In the clinical history median time of immediate reactions (76 cases, 81%) between drug intake and bronchospasm was 20 minutes [15-30 minutes]. Median time of nonimmediate reactions (18 cases, 19%) was 120 minutes [120-390 minutes]. There were 50 oral provocation tests performed, 14 of them (28%) were positive. Median time between drug intake and immediate reactions (8; 57% of cases) was 22.5 minutes [20-30 minutes] and median time of nonimmediate reactions (6; 43% of cases) was 167.5 minutes [125-206.25 minutes]. Time delay between drug intake and bronchospasm in the clinical history and after oral provocation test was not statistically different.

## 

Over the last few decades drug hypersensitivity and asthma has become a growing public health problem. The incidence of aspirin induced asthma (AIA) in the normal population is 0.3 ± 0.6% [1,2]. Despite a wealth of literature about AIA, controversy remains as to its prevalence, with published data ranging from 4 to 44%, regardless of whether the patients had a history of aspirin induced asthma or markers for an increased risk of the syndrome [3]. Asthma is more severe in patients with AIA than in those without aspirin sensitivity [4]. Bronchoconstriction may be severe and life-threatening, requiring hospital admission, and, at times, requiring mechanical ventilation. Up to 25% of hospital admissions for acute asthma requiring mechanical ventilation may be because of nonsteroidal antiinflammatory drugs (NSAID) ingestion [5].

The diagnosis of aspirin intolerance was based on a typical history confirmed by positive aspirin provocation tests [6]. Differences in populations studied, methods used, definitions of outcomes, and criteria for defining sensitivity reactions may all be responsible for the variations in reported rates [7-9].

Patients with AIA begin to experience wheezing, nasal congestion, rhinorrhea, and dyspnoea after taking aspirin or other NSAID [3]. Other symptoms may include flushing, angioedema, and gastrointestinal distress. On the other hand, the initial presentation may be recurrent nasal polyps or chronic sinusitis [10].

Despite an extensive of literature about AIA a greater understanding of the problem is still desirable. As patients can not objectively estimate their health status, patient history data may be insufficient or overestimated.

The primary end point of this study is to assess the time between drug intake and drug induced hypersensitivity reaction in patients sensitive to NSAID in the clinical patient history and the relationship to oral provocation tests. Another primary end point is to evaluate the clinical symptoms of hypersensitivity to NSAID and the risk of bronchospasm induced the same chemical structure drugs in aspirin sensitive patients.

## Design of the study and methods

### Patients

Our study was performed in the Center of Pulmonology and Allergology of Vilnius University hospital Santariskiu klinikos. We included 76 patients with a history of hypersensitivity reactions to NSAID documented by the referring physician. Median age of patients was 48 years [35.0-60.0], range 21-77 years, 62 (81.58%) of them were women, and 25 (33.89%) patients reported having atopy. Asthma was established for 25 (32.89%) patients (asthma was diagnosed according to the criteria of the Global Inititiative for Asthma [GINA]),[11] 17 (22.36%) had AIA (according recurrence of the same clinical reactions to NSAID or positive oral provocation tests to NSAID in their clinical history) (Table 1).

**Table 1:** Patient Characteristics

Characteristics	Patients With Suspected Hypersensitivity
Patients	
N	76
Sex	
Male	14 (18.4%)
Female	62 (81.6%)
Age (years)	
Median 25-75 percentile	48 [35-60]
Patient history	
Bronchial asthma	25 (33.89%)
AIA	17 (22.36%)
Atopy	25 (33.89%)
Family history for allergies	21 (27.63%)

The patients had many different types of allergic reactions to NSAID in their history such as bronchospasm, rhinoconjunctivitis, laryngeal edema, urticaria, maculopapular eruption, anaphylaxis, anaphylactic shock. Dyspnoea (wheezing with bronchospasm) after ingestion of NSAID were verified by spirometry and considered as AIA. Cutaneous adverse drug reactions (skin reactions) were variable: pruritus, urticaria and/or angioedema, maculopapular and/or purpuric skin eruption, erythroderma (exfoliative dermatitis), hypersensitivity syndrome or drug reaction with eosinophilia and systemic syndrome (DRESS), generalized or localized eczema, systemically induced contact dermatitis, acute generalized exanthematic pustulosis (AGEP), purpura, leucocytoclastic vasculitis, Stevens-Johnson syndrome, toxic epidermal necrolysis (Lyell syndrome), fixed drug eruptions (FDE), or eczematous photosensitivity reactions [12]. When bronchospasm or skin reactions were associated with any other symptom, the patient was classified as "anaphylaxis" or "anaphylactic shock" if there was a drop in blood pressure as recently proposed.

## Methods

After resolution of clinical symptoms (at least 4 weeks), all patients underwent the standardized ENDA (*European Network of Drug Allergy*) diagnosis for drug allergy that included the ENDA questionnaire and provocation tests [13,14]. The standardized ENDA questionnaire lists 43 symptoms possibly related to drug hypersensitivity and the time delay between the administration of the drug and the reaction. The symptoms (cutaneous, respiratory, gastrointestinal, cardiovascular, psychologic, and also involvement of other organ systems) were listed. The questions about patient demographics, comorbidities, atopy, family history, a type of hypersensitivity reactions were included. From a clinical point of view, reactions can be classified into 2 groups: immediate reactions, appearing no more than 1 hour after drug intake and nonimmediate reactions. The nonimmediate reactions occur with variable intervals ranging from 1 hour to a few days (usually 24-48 hours). If symptoms such as dyspnea or cough appeared, the measurement of lung function was performed. All subjects involved in the study gave their written informed consent.

Patients were free of infectious disease, fever, or inflammatory reactions at the time of testing. If the drug to be tested induced an anaphylactic reaction, then the intake of *β*-adrenergic blocking agents would be discontinued (usually for 48 hours), as these drugs may interfere with the treatment of a possible systemic reaction elicited by test. Antihistamines were stopped for 3-5 days, glucocorticosteroids used for short-term treatment in high doses (> 50 mg daily) were stopped for a week, in low doses, for 3 days before the tests. Certain antidepressants (imipramines, phenothiazines) also had to be discontinued for 5 days before the testing according to allergological rules [15].

Oral provocation tests were performed for the patients with suspected NSAID according to the European Network of Drug Allergy/*European Academy of Allergy and Clinical Immunology *recommendations [15]. The oral drug provocation tests consisted of ingesting increasing doses of the suspected causal drug. One hundredth of the therapeutic dose of suspected drug was administered as an initial dose, and was increased once every 30 minutes until the usual daily dose was reached or until symptoms of a drug reaction occurred (Table 2). Administration was single-blinded and performed by a physician with full resuscitation back-up on the ward. The risk-benefit analysis was made by the allergist with regards to the clinical reaction, the possibilities of treatment for a possible adverse reaction, the risk for the patient and the importance of the drug. The suspected drug was initially tested at a higher dilution of the test preparation (eg, 1/10-1/100000) for the patients with a history of anaphylactic shock. The patients were observed for the after reactions: respiratory (bronchospasm, tightness of chest, wheezing), nasal (rhinorrhoea, nasal congestion), and general reactions (ocular injection, periorbital swelling, skin reactions). Pulmonary function tests (forced expiratory volume in 1 second [FEV1], forced vital capacity [FVC]), and arterial blood pressure, observation of the patient were carried out every 30 minutes until the daily dose was reached and up to 48 hours after the last dose of a drug. The oral drug provocation test result was considered positive if any of the symptoms or signs of a previously described drug reaction were documented. The decrease of at least 20% in FEV1 observed during the test or till 3 hours after the last drug dose intake was considered as a bronchospasm. The oral drug provocation test result was considered negative if no sign of drug hypersensitivity occurred after the usual daily dose has been administered. Patients were kept under medical surveillance for 48 hours after the test procedure.

**Table 2:** Sequence of Increasing Drug Dosage During Drug Provocation Tests

Drug	Doses (mg)*
Aspirin	1, 5, 20, 50, 100, 200, 500
Paracetamol	1, 10, 50, 250, 500, 1000
Celecoxib	1, 10, 25, 50, 100
Diclofenac	1, 5, 20, 50
Ibuprofen	1, 5, 20, 50, 150, 200
Ketoprofen	1, 5, 20, 50
Nimesulide	1, 10, 20, 50, 100

*Ten times less than the first dose for anaphylactic shocks.

Bronchospastic and other clinical reactions after either challenge were relieved by short-acting *β*2-adrenomimetics, glucocorticosteroids, and antihistamines. No severe or long-lasting reactions were observed that could require a longer hospitalization or treatment in the intensive care unit. Lung function values (FVC, FEV1) were measured by MicroLab spirometer. The results of the oral provocation tests were compared with the results of patients' clinical history.

### Statistical Evaluation

Statistical evaluation was performed using PC and SPSS 17.0 software. Summary statistics were expressed as frequency, median, mean, SD. Because of the nonnormality of our data as determined by the Shapiro Wilk W Test, a global parametric ANOVA was avoided. Instead, a Mann-Whitney U Rank Sum Test was used to compare 2 in dependent groups; Wilcoxon's Signed Rank Test for matched pair studies, and a Kruskal-Wallis test and Friedman's Rank ANOVA for comparison of several groups were used. The relationship between variables was determined with Sperman's Correlation Coefficient.

## Results

There were 154 cases of hypersensitivity reactions to NSAID recorded in the clinical history. The most often used NSAID was aspirin. It induced 36 (22.93%) hypersensitivity reactions in the patients' clinical histories (Table 3). There were noticed clinical symptoms (separately or in combination) in the patients' history: 87 (56.49%) bronchospasms, 128 (83.11%) skin reactions, 88 (57.14%) anaphylaxies, and 11 (7.14%) anaphylactic shocks. There were 27 patients (35.52%) who reported having monosensibilization to NSAID and 49 patients (64.47%) had reactions to several NSAID and other chemical groups of drugs.

**Table 3:** The Frequency of Drug Induced Clinical Manifestations

Drug	Number of Clinical Histories	Percentage
Aspirin	36	22.93
Aspirin and Paracetamol (in combination)	22	14.01
Pyrazolone	20	12.74
Diclofenac	18	11.46
Paracetamol	16	10.19
Ketorolac	14	8.92
Ibuprofen	10	6.37
Other	21	13.38

In the clinical history the most frequent reactions with bronchospasm after use of NSAID were immediate reactions (79 cases, 81%; *P *< 0.05) with median time between drug intake and bronchospasm about 20 minutes [15-30 minutes]. Median time of nonimmediate reactions (19 cases, 19%) was 120 minutes [120-390 minutes].

### Oral Provocation Test Results

There were 50 (65.79% of all the patients) oral provocation tests with NSAID (Table 4) performed. Twenty six (34.21%) patients with an unequivocal history of aspirin or other NSAID intolerance, testing was not carried out because of clinical instability or lack of patient consent. Aspirin was the most often used drug in the oral provocation tests (16 cases, 32%). The true hypersensitivity to aspirin was in half of tested patients (Table 4); 14 (28.00%) of all oral provocation tests were positive. The most frequent hypersensitivity reaction was skin reaction, 8 cases of all oral provocation tests (16.00%, *P *< 0.001), while rhinitis or rhinoconjuctivitis occurred in 6 cases (12.00%, *P *< 0.001). Bronchospasm was observed only in 5 cases (10.00%, *P *< 0.001) separately or in combination with other clinical signs. In 3 cases, bronchospasm occurred in the provocation test with aspirin, in 1 case with paracetamol, and 1 with pyrazolone. The highest sensitivity was observed for aspirin, 8 cases (16.00%) out of all hypersensitivity reactions (Table 4).

**Table 4:** Oral Provocation Test Drugs

Drug	Number of the Oral Provocation Tests (%)	Number of Positive Cases (%)
Aspirin	16 (32.00)	8 (57.14)
Nimesulide	9 (18.00)	2 (14.29)
Paracetamol	5 (10.00)	1 (7.14)
Diclofenac	5 (10.00)	1 (7.14)
Other	15 (30.00)	2 (14.29)

**Figure 1:**
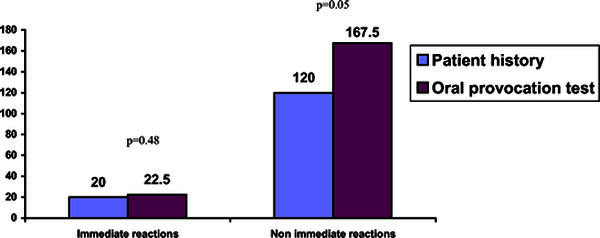
**The time delay frequency of immediate and nonimmediate reactions according to the patient's history and oral provocation test**.

A rapid answer to NSAID during the provocation tests was more common with 8 (57.14%) cases for immediate reactions. There were observed nonimmediate reactions in 6 (4.86%) cases (*P *< 0.05). Median time between drug intake and immediate reactions in the oral provocation test was 22.5 minutes [20-30 minutes] and median time of nonimmediate reactions was 167.5 minutes [125-206.25 minutes] (Table 5, Figure 1).

**Table 5:** The Time Delay Until Bronchospasm in the Clinical History and After the Oral Provocation Test

	Patients' History	Oral Provocation Test
Reaction type n (%)		
Immediate type (< 1 hour)	79 (81)	8 (57)
Nonimmediate type (> 1 hour)	18 (19)	6 (43)
Median time delay (minutes) 25-75 percentile		
Immediate type	20 [15-30]	22.5 [20-30]
Nonimmediate type	120 3[20-390]	167.5 [125-206.25]

## Discussion

The prevalence of AIA is still controversial. In adult asthmatics, it ranges 3 ± 21% depending on the diagnostic methods used [3]. Analyses based on the use of a questionnaire resulted in a higher number of positive results than did retrospective analyses of medical records. Prevalence rates of 11-24% were presented in 4 studies where questionnaires [16-19] where used and only 2-3% were obtained relying on medical records in 2 studies [20,21]. When aspirin challenge was coupled with spirometry, the frequency among adult asthmatics was 8 ± 20%, whereas surveys relying on history alone have reported a lower frequency, usually ~5% (7 ± 10) [5]. It is remarkable that 15% of patients were completely unaware of being aspirin-intolerant and realized that only after performance of provocation tests. According to Samp-son,[22] the reasons for underreporting of aspirin sensitivity may include the deliberate avoidance of NSAID by asthmatics aware of the risk of adverse reactions, or a lack of recognition by patients of mild NSAID-induced reactions because of their delayed onset of action. Underdiagnosis of aspirin sensitivity may be because of the lack of routine aspirin challenge testing of asthmatic patients because they do not report a positive history of aspirin sensitivity. On the other hand, intolerance to aspirin can be masked by such drugs as corticosteroids or long-acting *β*2-mimetics [23].

In the first step of our study, we compared patient history information with results of the oral provocation tests. Oral drug provocation test is widely considered to be the "gold standard" to confirm or refute the diagnosis of drug hypersensitivity to a certain substance as it can reproduce allergic symptoms and any adverse clinical manifestation irrespectively of the mechanism [15]. Oral challenge tests with aspirin were introduced systematically into clinical practice in the early 1970s,[24] and in consecutive years they were validated by Stevenson, Simon,[25] Dahlen, Zetterstroem,[26] Nizankowska-Mogilnicka,[27] and ENDA group of EAACI [13]. In our study, we performed the oral provocation test according to the ENDA recommendations [13,14].

The diagnosis of NSAID intolerance was based on a typical history, confirmed by positive oral provocation tests, which were carried out for 65% and were positive in 28% of patients in our study. Usually, the diagnosis of NSAID hypersensitivity is based only on history, but it is a vague and unreliable indicator. In fact, 55% of all the patients who previously labeled as sensitive to NSAID have tolerated NSAID when assessed by oral challenge, whereas 13.8% were truly NSAID sensitive in Schulert et al study [27].

After oral provocation tests in our small study we found that 28% of patients were sensitive to NSAID. The results were similar to the results of Jenkins, who showed 21% sensitivity to NSAID in asthmatic [3]. Some of them were unaware of their sensitivity because either they have never taken aspirin or they developed AIA in adulthood after years of apparent tolerance. Because aspirin and other NSAID are often self prescribed, patients with asthma should be alerted to the possibility of cross reaction between the drugs [3]. Sensitivity to aspirin itself was confirmed the most frequent in our study (57% of patients with aspirin related clinical history) as large, and the Demoly et al study with 47% positive tests results to aspirin [29]. In the same study, Demoly [29] showed that skin reaction, especially urticaria and maculopapular exanthema occupied an important place in the clinical history and dominated the reaction response after provocation tests. The most common hypersensitivity reaction in patient history and after oral provocation tests was skin reaction and it was found in 83% of all tested patients. Sánchez-Borges et al showed the same results, 86% of cutaneous pattern in the clinical patient history [30]. In our study, only 28% were confirmed sensitivity to NSAID after drug provocation test, skin reactions (16%) were the most frequent as in the clinical history. Our data were similar to Schubert et al,[28] who found that 61.5% of 260 patients tested described their NSAID hypersensitivity as cutaneous reactions (urticaria, angioedema), 24.2% as respiratory symptoms (asthma, rhinitis), 3.5% as anaphylactic reactions, 10.8% described uncertain signs.

Comparing with patient history results (56.5% of bronchospasm in our patients), true bronchospasm is statistically significant less common as skin reactions or rhinitis and we had only 10% patients developed this clinical symptom (*P *< 0.01) in our study. The percentage of patients with decrease of FEV1 after provocation test in our investigation was less significant as the Williams et al study with 35% patients reacted with bronchospasm and more than half of them developed severe dyspnoea [31]. The results show that the risk of bronchospasm after NSAID ingestion was not significant, but can be severe.

According to the literature, median time delay is 30 minutes to 3 hours after taking aspirin or other NSAID [3,4]. We found that immediate type of hypersensitivity reactions was significantly more frequent in the clinical history and in oral provocation tests than the nonimmediate reactions (81 vs. 57%). Our study results show that median time delay of bronchospasm in clinical history and after test (20 vs. 22.5 minutes, *P *= 0.481) was not statistically different. Nonimmediate reactions were quite rear in the clinical history comparing with the results of the oral provocation tests (19 vs. 43%). The median time delay between ingestion of NSAID and bronchospasm was 120 versus 167. 5 minutes (*P *= 0.05).

In summary, our data suggest that true hypersensitivity to NSAID rarely manifests as a bronchospasm. The time delay between drug intake and the bronchospasm in the clinical history and after oral provocation test was not statistically different. The clinical reactions in the history and after oral provocation test repeat the same symptoms. The patient history results show that patients obviously recognize the reaction time comparing with the oral provocation test results. More frequent number of immediate type of hypersensitivity reaction in the patient history may be because of patients' lack of knowledge about nonimmediate reaction type or because of late bronchospasm that might be reduced by maintenance therapy with additional controller therapy, including long-acting *β*-agonists.

## End Notes

Abstract of this article was presented at World Allergy Congress XXI, December 6-10th, 2009, Vilnius, Lithuania, (Number 0531).
